# The Roentgen Rays and Colour-Blindness

**Published:** 1899-05-20

**Authors:** 


### THE ROENTGEN RAYS AND COLOUR-BLINDNESS.

It is always well to clear up fallacies by actual experiment as quickly as may conveniently be done. Ever since the discovery of the Roentgen rays the assertion has again and again been made that by their aid the blind had been made to see, and all the rest of it, but none of the cases of alleged cure of blindness seems to have stood the test of critical examination. Lately, however, it has been claimed that the retinæ of colour-blind persons are acted on strongly by the Roentgen rays, but Dr. Sydney Stephenson and Dr. David Walsh,[1] after testing the matter experimentally on four colour-blind children, have come to the conclusion that there is no reason to suppose that the retinæ of these children reacted to the focus tube in any way differently from those of folk with normal colour sense.

[1] Lancet, May 13.

